# TUG1 enhances high glucose-impaired endothelial progenitor cell function via miR-29c-3p/PDGF-BB/Wnt signaling

**DOI:** 10.1186/s13287-020-01958-3

**Published:** 2020-10-15

**Authors:** Yang Li, Kangkang Zhi, Shilong Han, Xue Li, Maoquan Li, Weishuai Lian, Haijun Zhang, Xiaoping Zhang

**Affiliations:** 1 Department of Interventional & Vascular Surgery, Tenth People’s Hospital of Tongji University, Shanghai, 200072 China; 2 Institute of Interventional & Vascular Surgery, Tongji University, Shanghai, 200072 China; 3 Department of Vascular and Endovascular Surgery, Changzheng Hospital, Shanghai, 200003 China

**Keywords:** lncRNA taurine upregulated gene 1, Platelet-derived growth factor-BB, Endothelial progenitor cells, Diabetes, Angiogenesis

## Abstract

**Background:**

Diabetes is associated with the dysfunction of endothelial progenitor cells (EPCs), characterized as impaired angiogenesis, a phenomenon thought to be involved in the development of diabetic foot. lncRNA plays an essential role in microvascular dysfunction and signaling pathways in patients with diabetes. lncRNA taurine upregulated gene 1 (TUG1) participates in angiogenesis in various cells. However, the mechanisms of TUG1 activity in EPCs have not been elucidated.

**Methods:**

We isolated and then characterized EPCs from the peripheral blood of mice using immunofluorescence and flow cytometry. Western blot detected the wnt/β-catenin pathway in high glucose-treated EPCs. Bioinformatics analysis predicted a putative binding site for TUG1 on miR-29c-3p. The interactions among TUG1, platelet-derived growth factor-BB (PDGF-BB), and miR-29c-3p were analyzed by luciferase assays. In vivo, diabetic mouse ischemic limb was treated with normal saline or TUG1 overexpression lentiviruses.

**Results:**

We found that EPC migration, invasion, and tube formation declined after treatment with high glucose, but improved with TUG1 overexpression. Mechanically, wnt/β-catenin pathway and autophagy were involved in the function of TUG1 overexpression in high glucose-treated EPCs. Moreover, TUG1 regulates the PDGF-BB/wnt pathway and function of high glucose-treated EPCs via miR-29c-3p. In vivo, injection of TUG1 lentivirus in a diabetic mouse ischemic limb model stimulated angiogenesis.

**Conclusions:**

Our findings suggest that TUG1 restores high glucose-treated EPC function by regulating miR-29c-3p/PDGF-BB/Wnt signaling.

## Introduction

Diabetic foot syndrome (DFS) is one of the most common complications of diabetes mellitus, both type 1 and type 2, and it is the leading cause of hospitalization in patients with type 2 diabetes [1]. For all patients with diabetes, the lifetime risk developing a foot ulcer is 25%, and the majority of these will advance to amputation within 4 years of diagnosis [2]. The etiology of DFS is multifactorial. Risk factors include diabetic neuropathy and peripheral vascular disease [3]. However, it remains unclear as to whether there is a common etiological factor giving rise to DFS. Several new compelling lines of evidence suggest that the final common etiology may involve stem cell dysfunction, particularly endothelial progenitor cells (EPCs) [4]. Bone marrow-derived EPCs are capable of proliferating, migrating, and differentiating in response to specific stimuli (i.e., hypoxia), making them vital roles in processes such as angiogenesis and post-ischemic neovascularization [5–8]. Hypoxia is pathologically associated with diabetes, but a study indicated that EPCs are significantly decreased (40%) in diabetic patients with peripheral vascular disease [9]. EPCs, when recruited, migrate in the bloodstream as circulating progenitor cells (CPCs), and this recruitment is also highly affected in diabetes [9, 10]. In addition, high glucose concentrations increase apoptosis and decrease the proliferation of CPCs, which in turn contributes to slow wound healing in diabetic patients [10]. Many studies on animal models and humans agree that EPCs and CPCs are highly affected in diabetes, and proper migration, differentiation, and proliferation of EPCs are vital for vascular neogenesis in DFS [10–12]. A growing body of evidence suggests that mesenchymal stem cell therapy may be used to accelerate wound healing in DFS [13]. Nevertheless, the mechanisms of native stem cell dysfunction in the context of DFS have not been clearly elucidated.

Recent advances in cell biology may provide clues to the pathogenesis of DFS. Long non-coding RNAs (lncRNAs) are non-translated nucleic acid elements that participate in a number of regulatory mechanisms both in normally functioning and in diseased cells and tissues [14–17]. Whereas lncRNAs do not themselves code for proteins, they participate in epigenetic processes by specific binding to other nucleic acid-containing elements, including microRNA (miRNA). Several lncRNAs have been implicated in the pathology of diabetes mellitus as well as its complications [18]. One lncRNA in particular, taurine upregulated gene 1 (TUG1), is involved in several biophysical processes, including angiogenesis [19–21]. One of the mechanisms of action of TUG1 is its binding to miRNAs, which are small (~ 22 nucleotide) elements involved in post-translational modification of gene expression and RNA silencing. One of these miRNAs, miR-29c-3p, is involved in metastasis and proliferation of melanoma cells [22]. Despite the broadening fund of knowledge regarding the action of TUG1 and miR-29c-3p in the regulation of angiogenesis, and despite the growing body of evidence to suggest that impaired angiogenesis is involved in the pathophysiology of DFS, little is known regarding the role of TUG1 and miR-29c-3p in EPC function in diabetes.

In the present study, we applied high glucose to induce diabetes internal environment. Our study investigated the effect of TUG1 on high glucose-treated EPC function and its underlying mechanism of angiogenesis. Further, we evaluated the pathophysiology of impaired angiogenesis in vivo models of diabetes.

## Materials and methods

### Animals

Male 8-week-old C57BL/6 J mice were acquired from Shanghai Laboratory Animal Center Ltd., China. Food and water were provided ad libitum. The animals were maintained on a 12-h light/dark cycle.

### Isolation and characterization of EPCs

EPCs were isolated and characterized as previously described [23]. Briefly, crude cells were isolated from mouse bone marrow and cultured in a 37 °C incubator under 5% CO_2_. We used passages 3–7 for experiments. EPCs were characterized using immunofluorescence. Briefly, cells were fixed in 4% paraformaldehyde (PFA), incubated with Human Dil-Acetylated Low Density Lipoprotein (Dil-Ac-LDL) and FITC-labeled *Ulex europaeus* agglutinin 1(FITC-UEA-1) (Sigma Deisenhofen, Germany), and visualized using a confocal microscope. We further characterized EPCs using flow cytometry. Positive cells were identified using antibodies against CD34, CD133, and VEGF-2 (all the antibodies were purchased from BD Bioscience, USA).

### Cell treatment

The TUG1 overexpression vector, miR-29c-3p mimic, and vehicle were purchased from GenePharm Co. Ltd. (Shanghai, China). Twenty-four hours after transfection, cells were collected for analysis. For high glucose treatment, EPCs were cultured in serum-free DMEM containing 25 mM high glucose for 24 h [24]. For Wnt pathway and autophagy experiments, EPCs were incubated with Wnt pathway inhibitor Dickkopf-1(DKK1) (0.1 mg/ml) [25] and autophagy inhibitor 10 μM chloroquine (CQ) (Sigma-Aldrich) [26].

### Immunofluorescence

EPCs were seeded in six-well plates and fixed in 4% PFA. For tissue immunofluorescence experiments, gastrocnemius muscle tissue was isolated from ischemic hind limbs, fixed in 4% PFA, embedded in paraffin, and then cut into 5-μm sections for immunofluorescence staining. Sections were blocked with 10% bovine serum albumin and incubated with primary antibody. This was followed by incubation with fluorescently labeled secondary antibodies. Nuclei were labeled with 4′,6-diamidino-2-phenylindole (DAPI) (Beyotime, Shanghai), and cells were visualized using fluorescence microscopy.

### Wound scratch assay

EPCs were seeded in six-well plates. When EPCs are spread over the entire six-well plate, the medium was removed, and a 200-μl pipette tip was used to create a linear scratch. Then, EPCs were washed twice with PBS and treated with 25 mM high glucose or overexpression of TUG1 for 24 h. Images were obtained by an Olympus inverted phase-contrast microscope. The number of migrating cells was evaluated by cell counter counting the migrated cells in three random microscopic fields.

### Transwell assay

EPCs (1 × 10^5^) were plated in the upper portion of transwell chambers (8 μm, 24-well plates) that had been coated with Matrigel [27]. The insert membranes were cut out and stained with crystal violet (Beyotime Technology, China), and invading cells were photographed and counted using an inverted phase-contrast microscope.

### Tube formation assay

EPC tube formation was assessed using an in vitro Angiogenesis Assay Kit (Chemicon, Billerica, MA, USA) as described previously [25]. EPCs (1 × 10^4^) were grown in EC matrix solution with EGM-2 MV medium, after which the number of tube formation in the matrix gel was examined under a microscope after 16 h of incubation. Tube formation was evaluated under an inverted light microscope. The area of tube formation was quantified in three random fields; the total area of tube formation represented the degree of angiogenesis.

### Ischemic hind limb model construction

For construction of the ischemic hind limb model, diabetes mice were used [28]. To create the model, mice were anesthetized and the hind limb area was depilated. The proximal and distal portions of the femoral artery were ligated. After surgery, buprenorphine (0.04 mg/kg) was injected to reduce the pain. Normal saline or TUG1 overexpression lentiviruses were injected into the distal ischemic hind limb. Four weeks later, hind limb blood perfusion was measured using a Laser Doppler perfusion imager system (Moor Instruments Limited, Devon, UK). Red sections indicated richer perfusion.

### Dual-luciferase reporter assay

The Dual-Luciferase Reporter Assay System (Promega, Madison, WI, USA) was used [29], and segments of TUG1 and the 3′-untranslated region (UTR) of platelet-derived growth factor type BB (PDGF-BB), containing miR-29c-3p binding sites (WT) or mutated binding sites (MUT), were synthesized by Sangon Biotech (Shanghai, China). These fragments were subcloned into the pGL4-Basic vectors (Promega). Cells were cotransfected with constructs containing WT or MUT TUG1 and the 3′-UTR of PDGF-BB and miR-29c-3p mimics. After 48 h, cells were harvested, lysed, and measured using a plate reader.

### Real-time reverse-transcription polymerase chain reaction (qRT-PCR)

Total RNA was isolated from cells using TRIzol reagent (Invitrogen, Carlsbad, CA, USA), according to the manufacturer’s protocol. Reverse transcription was carried out using a Prime Script RT reagent kit (TaKaRa, Dalian, China), according to the manufacturer’s instructions.

Amplification was carried out using SYBR Premix Ex Taq (TaKaRa). For miR-29c-3p, qRT-PCR was performed using a microRNA assay kit. Relative gene expression was calculated using the 2^˗ΔΔCT^ method. PCR reactions were performed in triplicate, using the following primers—PDGF-BB Forward: CAGTGACCTTGGAGGACCAC, Reverse: GAATGGTCACCCGAGCTT-GA; TUG1 Forward: CTGAAGAAAGGCAATCCATC, Reverse: GTAGGCTACTACAGGTCATTTG; and GAPDH Forward: GTGAAGGTCGGAGTCAACGG, Reverse: TCCTGGAAGATGGTG-ATGGG.

### Western blot analysis

EPCs were harvested in cold RIPA buffer, and the protein was extracted by protein extraction kit (Jiancheng, Nanjing) and quantified by a BCA assay kit (Solarbio Life Sciences). Proteins (20 μg per lane) were separated by 10% SDS-PAGE and were then transferred to PVDF membranes. Membranes were blocked with 5% non-fat dry milk and incubated with primary antibodies: Wnt (1:1000), p-β-catenin (1:1000), PDGF-BB (1:500), and LC3 I/II (1:1000) (all antibody purchased from Cell Signaling Technology) at 4 °C overnight. Then, membranes were washed in TBS-Tween five times, followed by incubation with HRP-conjugated secondary antibodies (1:5000) (Cell Signaling Technology) for 1 h at room temperature. GAPDH was used as the loading control. Bands were detected using an ECL assay kit. Protein gray values were measured using ImageJ software.

### Statistical analysis

All experiments were performed at least three times. Data were expressed as the mean ± standard deviation (SD). Comparisons were performed using unpaired Student’s *t* test. Data were analyzed using SPSS 21 (SPSS, Chicago, IL, USA). *p* < 0.05 was considered statistically significant.

## Results

### Identification of EPCs from mouse bone marrow

**Fig. 1:**
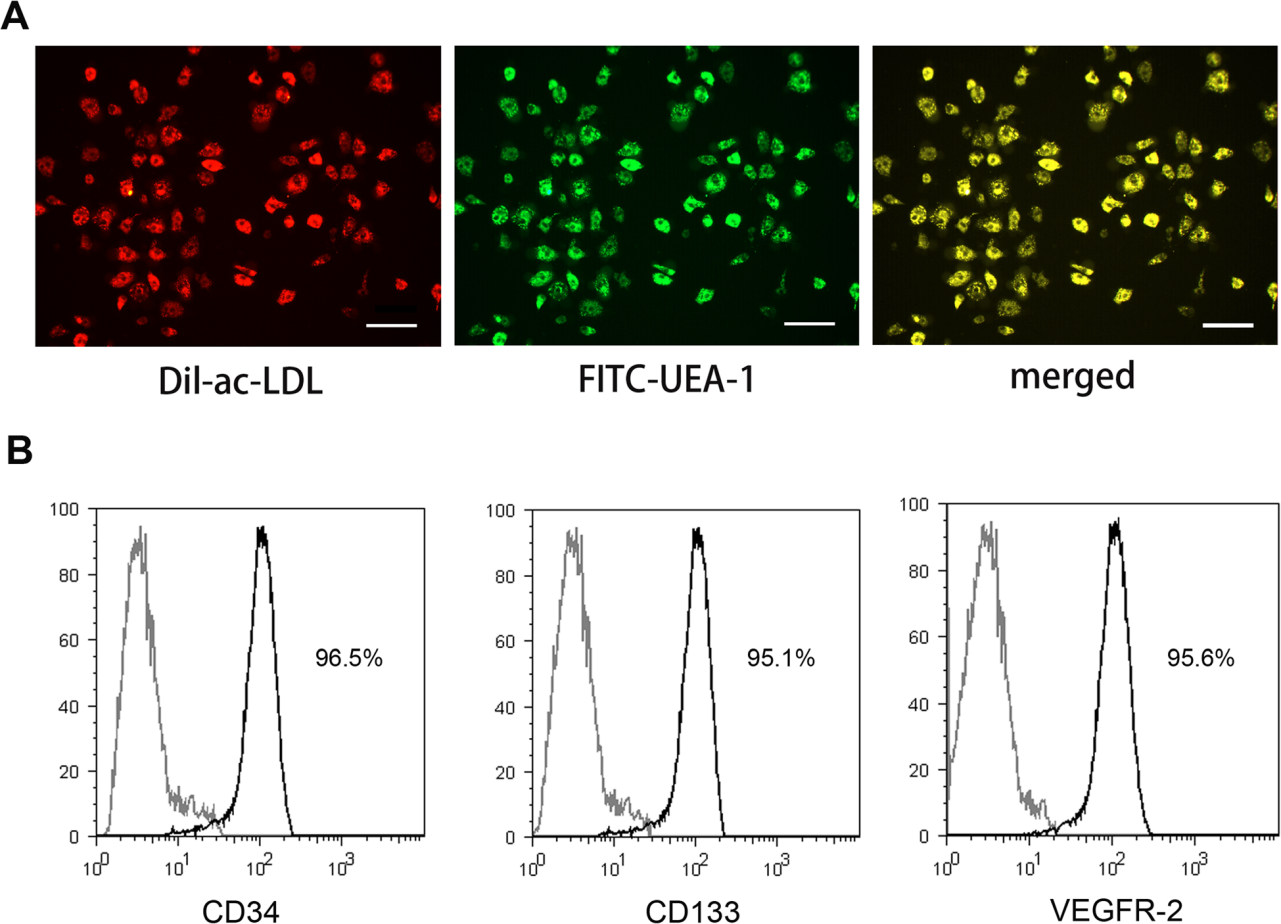
Identification of EPCs from mouse bone marrow. **a** Uptake of Dil-Ac-LDL and binding of FITC-UEA-1 are characteristic of endothelial progenitor cells. Scale bar = 50 μm. **b** Flow cytometry revealed positive expression of CD34, CD133, and VEGFR-2

Initially, we verified that the isolated EPCs exhibited the desired phenotype. Cells in the monolayers took up Dil-Ac-LDL and bound FITC-UEA-1, both of which are characteristics of EPCs. More than 90% of EPCs stained positive for Ac-LDL (red) and UEA-1 (green) (Fig. 1a). Flow cytometry was used to confirm the identity of these cells. We confirmed that the cultured cells expressed endothelial-specific markers such as CD34, CD133, and VEGFR-2, all of which are characteristic of EPCs (Fig. 1b).

### Overexpression of TUG1 restored the function of EPCs treated with high glucose

**Fig. 2:**
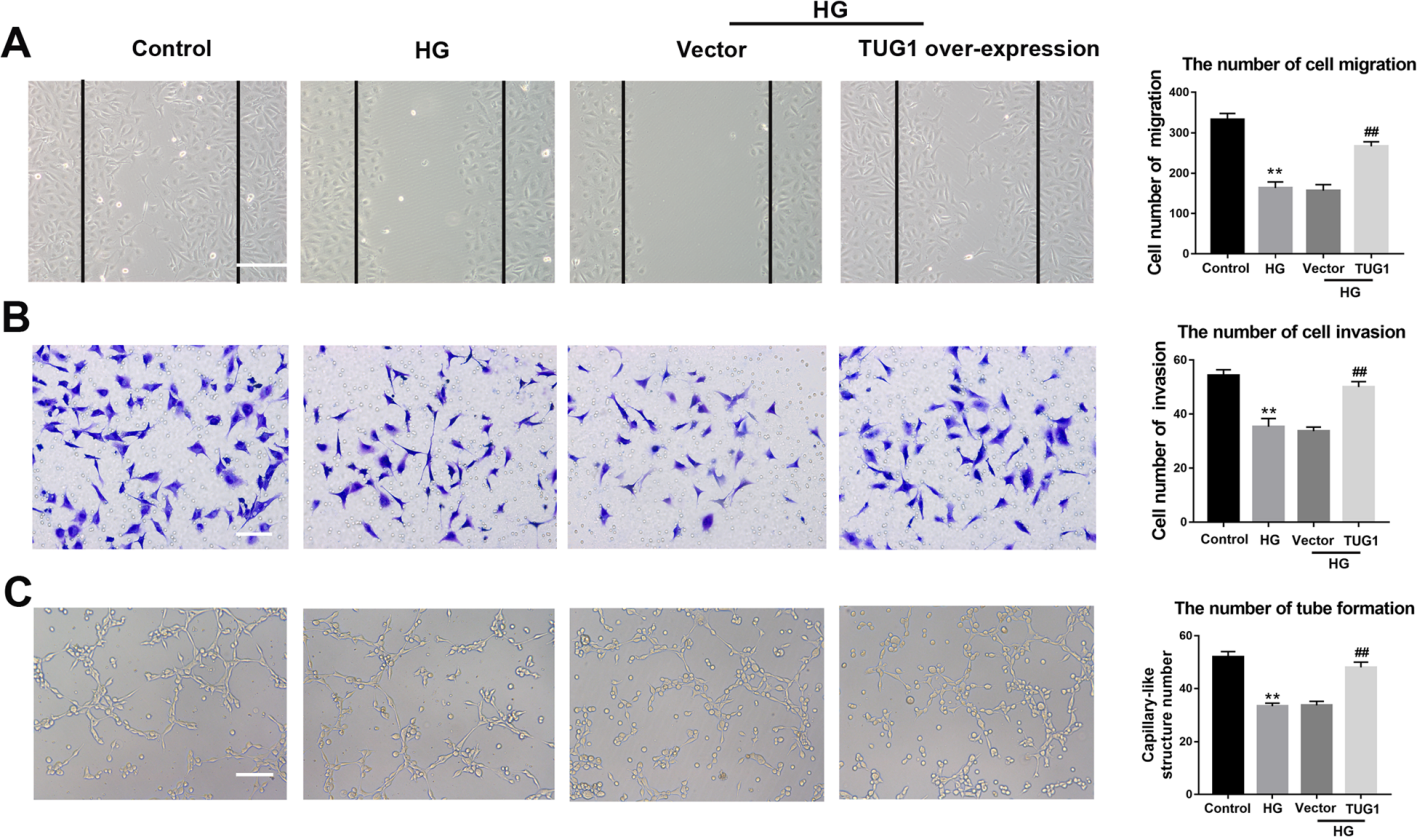
Overexpression of TUG1 restored the function of EPC treated with high glucose. **a** Wound healing assay showing the effects of overexpression of TUG1 on EPC migration, which was stimulated with high glucose (HG) (25 mM) for 24 h. Scale bar = 100 μm. **b** Cell migration was evaluated after overexpression of TUG1 on HG-stimulated EPCs. Scale bar = 50 μm. **c** Tube formation assay was evaluated after overexpression of TUG1 on HG-stimulated EPCs. Scale bar = 50 μm. **p < 0.01 vs control; ^##^*p* < 0.01 vs HG

As previously indicated TUG1 plays a vital role in angiogenesis, decreased expression of TUG1 was found in EPCs under high glucose (Supplementary Fig. S1). Hence, the effects of TUG1 overexpression on EPCs (Supplementary Fig. S2) in the presence of high glucose levels were tested using a wound healing assay, transwell migration assay, and tube formation assay (Fig. 2). The migration ability of EPCs in cells exposed to high glucose (25 mM) was compared with that of the control cells. In the wound healing assay (Fig. 2a), we found that TUG1 overexpression reversed the inhibition that was imposed by high glucose exposure. Consistent with this result, the transwell migration assay showed that TUG1 overexpression restored the migratory abilities of EPCs (Fig. 2b). Finally, tube formation was significantly greater in EPCs that overexpressed TUG1 than in high glucose-treated control cells or in cells transfected with blank vector (Fig. 2c). These findings suggest that hyperglycemia inhibits angiogenesis and that TUG1 expression antagonizes this inhibition.

### TUG1 promotes the expression of the PDGF-BB-wnt pathway in EPCs treated with high glucose

**Fig. 3:**
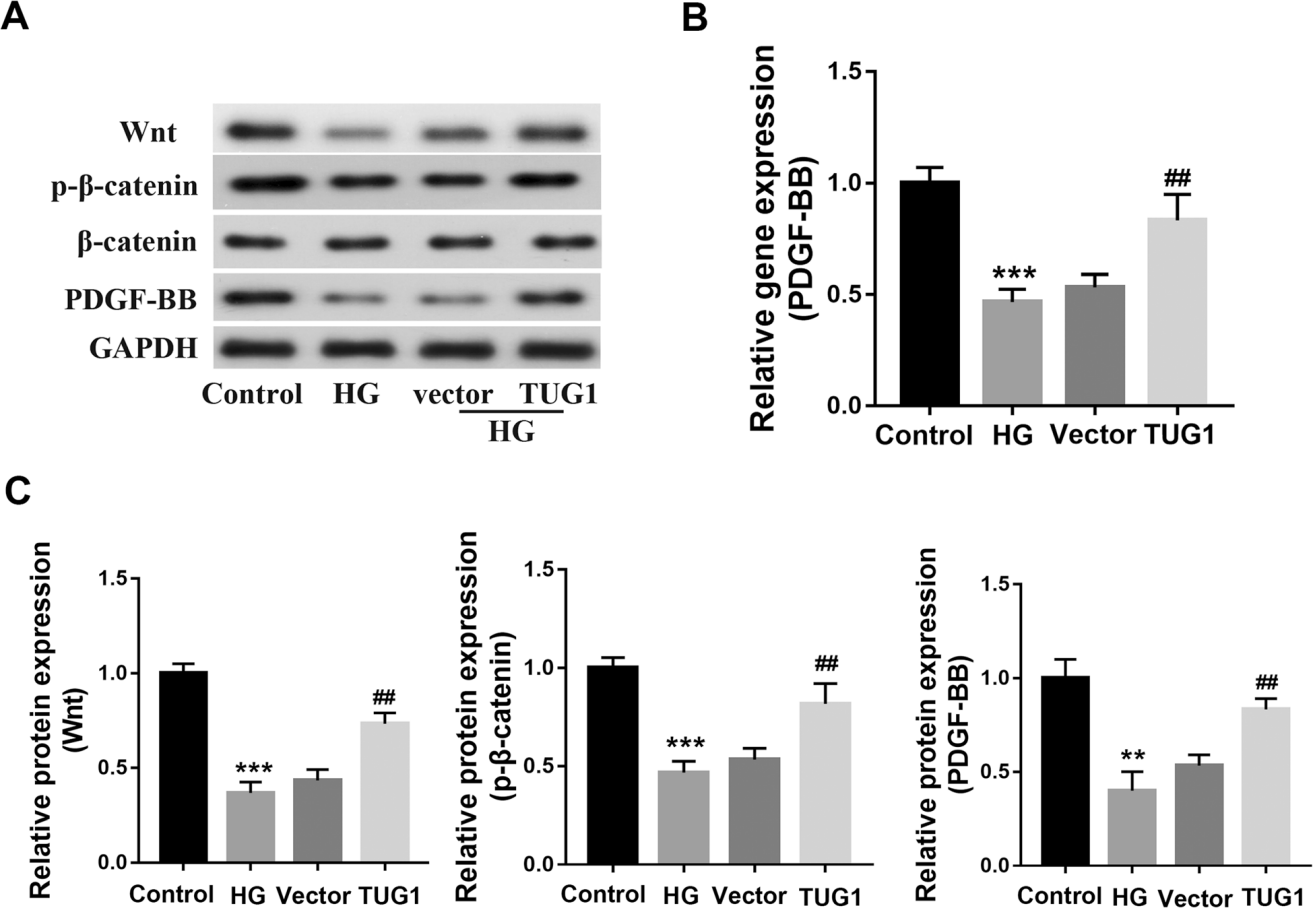
TUG1 promotes the expression of the PDGF-BB-Wnt pathway in EPCs treated with high glucose. **a** Western blot illustrating the levels of Wnt signaling pathway proteins and PDGF-BB after TUG1 overexpression in HG-treated EPCs. **b** qRT-PCR showing relative PDGF-BB RNA levels after TUG1 overexpression in HG-treated EPCs. **c** Relative quantification of protein levels of Wnt, p-β-catenin, and PDGF-BB under various conditions. **p < 0.01 vs control; ^##^*p* < 0.01 vs HG

To investigate the mechanism responsible for the effect of TUG1 overexpression on EPCs, the effects of transfected EPCs on expression levels of wnt pathway proteins were assessed (Fig. 3). Evidentially, high glucose medium (25 mM) inhibited the expression of PDGF-BB at the protein and mRNA levels, but that TUG1 overexpression reversed this inhibition (Fig. 3a, b). Similarly, high glucose exposure reduced the protein expression of wnt, p-β-catenin, and PDGF-BB in EPCs; however, overexpression of TUG1 largely reversed this inhibition (Fig. 3c). These findings suggest that the PDGF-BB and wnt pathway protein expression levels are impacted by the presence of hyperglycemia and that TUG1 functions to overcome these effects.

### Involvement of the wnt signaling pathway in the TUG1-mediated effects on high glucose-treated EPCs

**Fig. 4:**
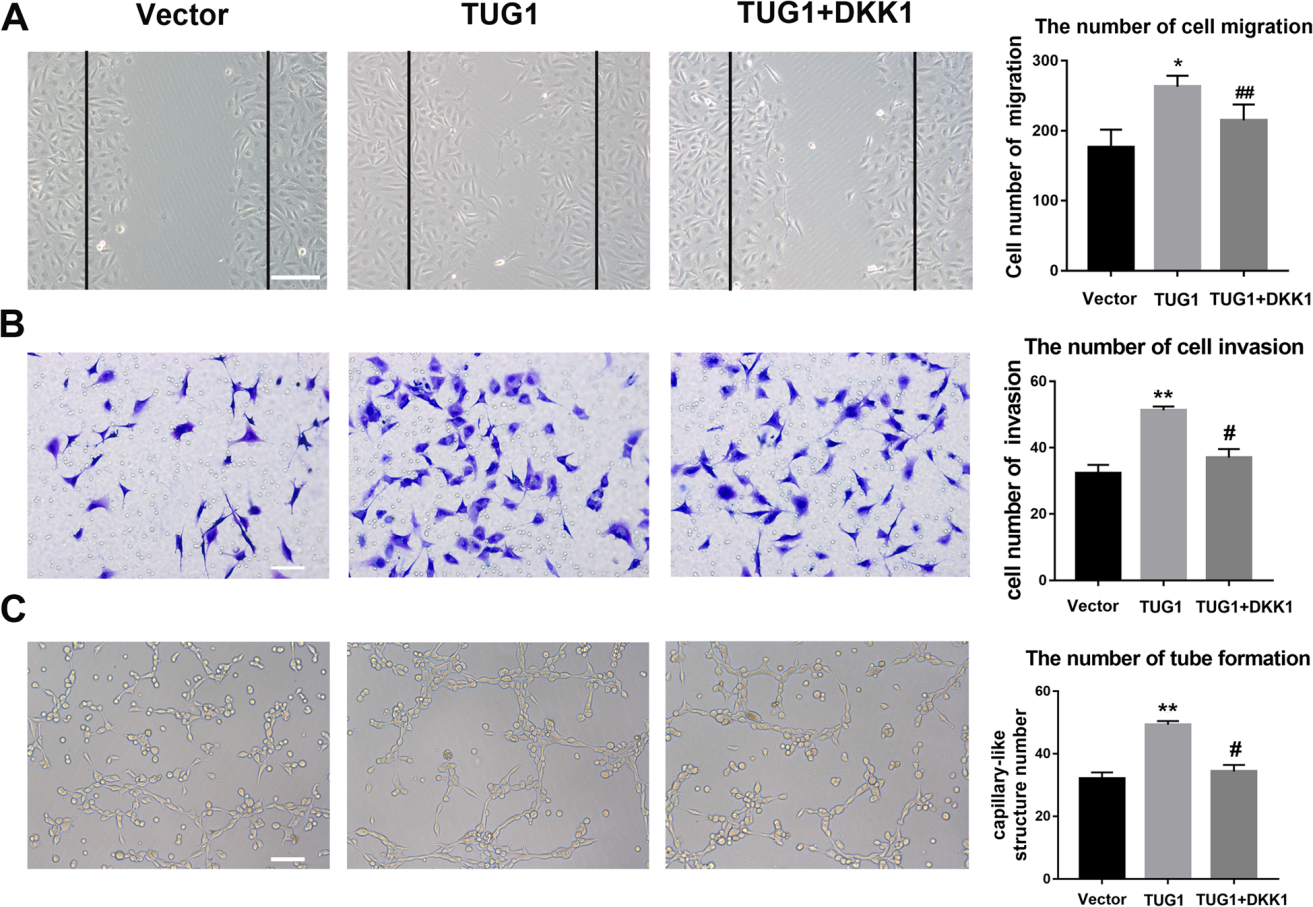
Involvement of the Wnt signaling pathway in the TUG1-mediated effects on HG-treated EPCs. **a** Wound healing assay showing the effects of the Wnt signaling pathway on EPCs after overexpression of TUG1. Scale bar = 100 μm. **b** Transwell cell migration assay provided results similar to those for wound healing. Scale bar = 50 μm. **c** Tube formation was evaluated after inhibition of the Wnt signaling pathway in EPCs. Scale bar = 50 μm. DKK1 = Wnt signaling inhibitor. **p < 0.01 vs vector; ^##^*p* < 0.01 vs TUG1

To further investigate the role of the wnt signaling pathway, wound healing, transwell migration, and tube formation assays were repeated in the presence or absence of DKK1, a wnt signaling inhibitor (Fig. 4). In EPCs exposed to high glucose levels, TUG1 overexpression restored wound healing, and DKK1 inhibited the effect of TUG1 (Fig. 4a). Similarly, in the transwell migration assay, treatment with DKK1 inhibited the restorative effect of TUG1 overexpression on EPC migration (Fig. 4b). Finally, in the tube formation assay, the addition of DKKI reversed the stimulatory effect of TUG1 overexpression (Fig. 4c). These data provided further evidence that the effects of TUG1 in correcting the effects of hyperglycemia on angiogenesis are mediated at least in part by the wnt pathway.

### Involvement of the autophagy pathway in the TUG1-mediated effects on high glucose-treated EPCs

**Fig. 5:**
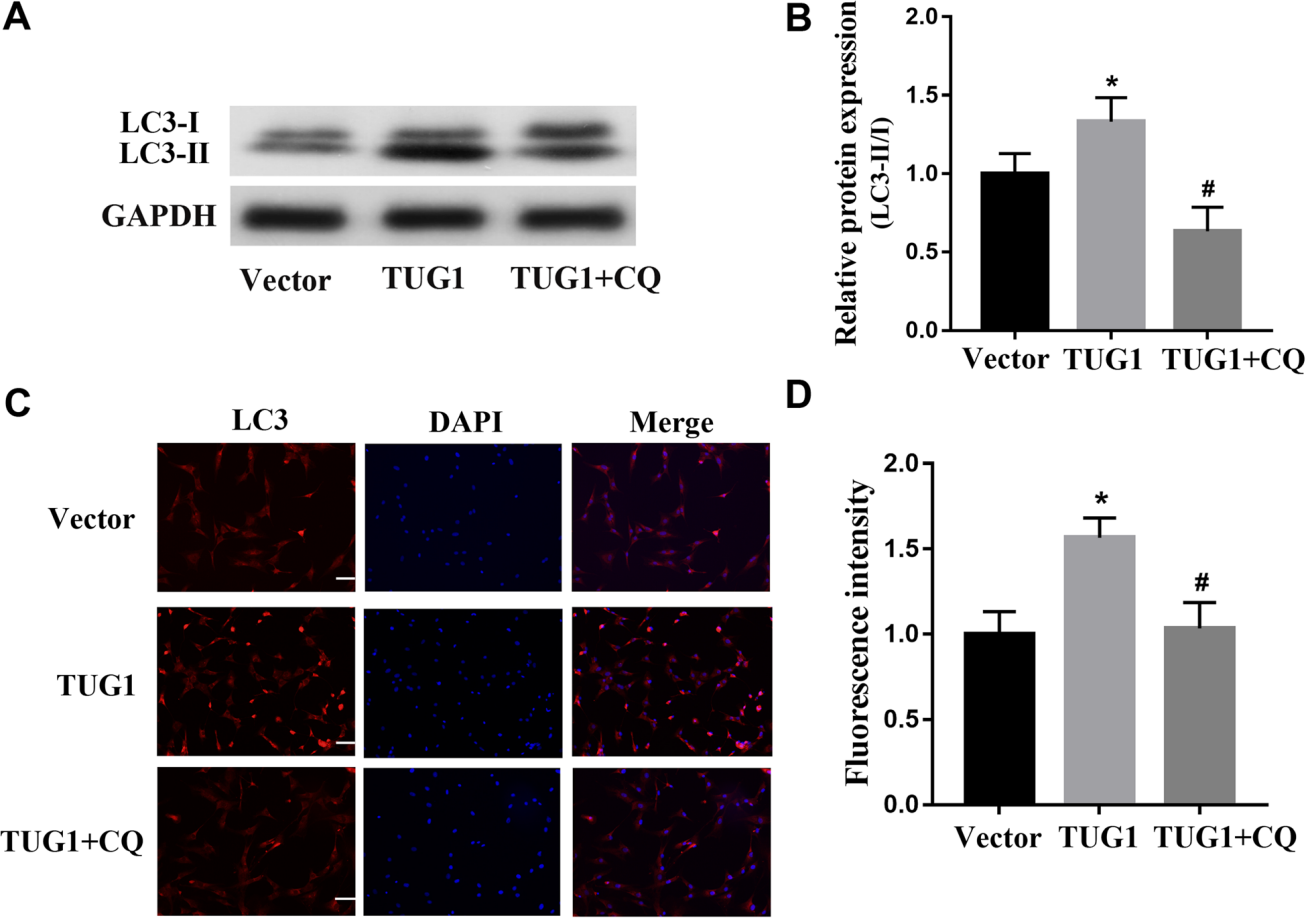
Involvement of the autophagy pathway in the TUG1-mediated effects on HG-treated EPCs. **a** Western blot analysis of LC3 levels after TUG1 overexpression or chloroquine (CQ) (10 μM) treatment in EPCs. **b** Relative quantification of LC3 protein levels after treatment with TUG1 overexpression or CQ on EPCs. **c** Immunofluorescence of LC3 protein (red) after treatment with TUG1 overexpression or CQ on EPCs. Scale bar = 50 μm. **d** Fluorescence intensity of LC3 protein (red) after treatment with TUG1 overexpression or CQ on EPCs. *p < 0.05, **p < 0.01 vs vector; ^##^*p* < 0.01 vs TUG1

The angiogenic behavior of EPCs is mediated, at least in part, by the autophagy pathway. To investigate the role of the autophagy pathway in the mechanism responsible for the TUG1-mediated effects on the function of EPCs, we used western blotting to measure expression levels of autophagy pathway proteins LC3I/II in the presence and absence of overexpression of TUG1 (Fig. 5). Clearly, TUG1 overexpression significantly increased the protein expression levels of LC3 and that autophagy inhibitor CQ blunted this effect (Fig. 5a, b). Immunofluorescence assays for LC3 were consistent with the western blot results (Fig. 5c, d). These data suggest that the effects of TUG1 on EPCs are mediated, at least in part, by the autophagy pathway.

### TUG1 regulates the PDGF-BB-Wnt pathway via miR-29c-3p

**Fig. 6:**
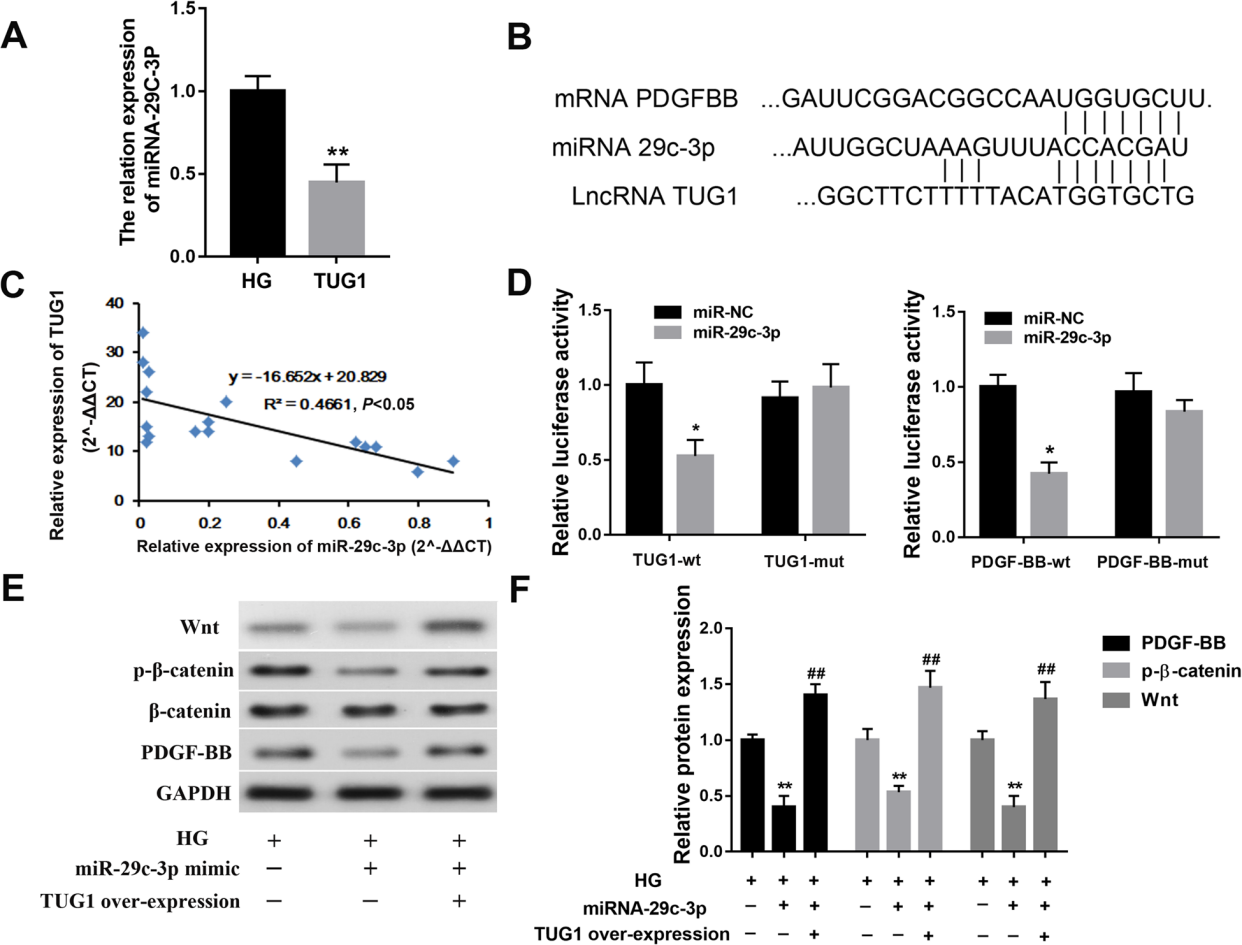
TUG1 regulates PDGF-BB-Wnt pathway via miR-29c-3p. **a** Real-time quantitative PCR confirmed the downregulation of miR-29c-3p in TUG1-overexpressing EPCs. **b** The predicted positions of miR-29c-3p binding sites in the PDGF-BB and TUG1 transcripts. **c** Correlation analyses indicated a negative linear association between the expression of TUG1 and miR-29c-3p. **d** Dual-luciferase reporter gene assay verifies the regulatory relationship between TUG1 and miR-29c-3p, PDGF-BB, and miR-29c-3p. **e** Western blot analysis of Wnt pathway and PDGF-BB in EPCs transfected with miR-29c-3p mimic and TUG1 overexpression lentivirus. **f** Relative quantification of protein levels of Wnt pathway and PDGF-BB in EPCs transfected with miR-29c-3p mimic and TUG1 overexpression lentivirus

We next explored the role of miR-29c-3p in the mechanism of TUG1 regulation of the PDGF-BB-wnt pathway (Fig. 6). Using real-time quantitative PCR, we found that EPCs overexpressing TUG1 had significantly lower expression levels of miR-29c-3p than the controls (Fig. 6a). Bioinformatics analysis revealed that there were miR-29c-3p binding sites in both the PDGF-BB and TUG1 transcripts (Fig. 6b). Correlation analyses suggested a negative linear association between the expression of TUG1 and miR-29c-3p (Fig. 6c). After transfection of miR-29c-3p mimic in EPCs, the expression level of miR-29c-3p increased significantly (Supplementary Fig. S3). Next, dual-luciferase reporter gene assays were performed to determine that there were indeed regulatory relationships between TUG1/PDGF-BB and miR-29c-3p (Fig. 6d–f).

### TUG1 regulates high glucose-impaired EPC function via miR-29c-3p

**Fig. 7:**
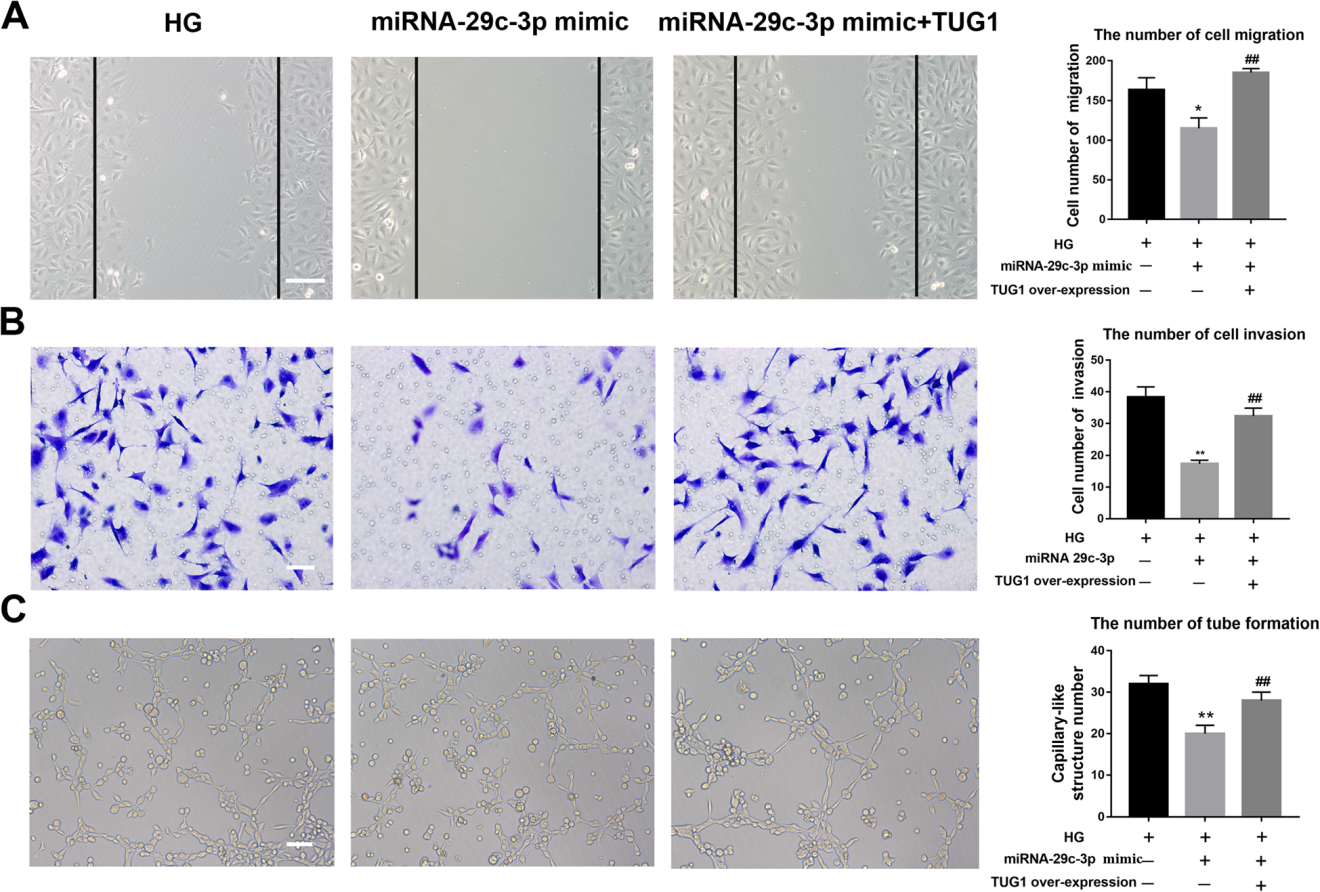
TUG1 regulates high glucose-impaired EPC function via miR-29c-3p. **a** Wound healing assay to study the effects of miR-29c-3p on EPC migration. Scale bar = 100 μm. **b** Transwell cell migration assay to determine the effects of miR-29c-3p on EPC invasion. Scale bar = 50 μm. **c** Tube formation assay to determine the effects of miR-29c-3p on EPC tube formation. Scale bar = 50 μm. *p < 0.05, **p < 0.01 vs HG; ^##^*p* < 0.01 vs HG+ miR-29c-3p mimic

Further in this study, the effects of TUG1 and miR-29c-3p expression on EPC function using migration, invasion, and tube formation assays were explored (Fig. 7). In the wound healing assay, it was clear that high glucose-exposed EPCs transfected with the miR-29c-3p mimic showed even less movement than cells exposed to high glucose alone, while the overexpression of TUG1 reversed the effects of the miR-29c-3p mimic transfection (Fig. 7a). Similarly, in the transwell migration assay, transfection further inhibited the movement of EPCs and TUG1 overexpression reversed this effect (Fig. 7b). Finally, in the tube formation assay, TUG1 reversed the inhibition of tube formation that was caused by the miR-29c-3p mimic transfection (Fig. 7c).

### TUG1 overexpression promotes angiogenesis in diabetic mice

**Fig. 8:**
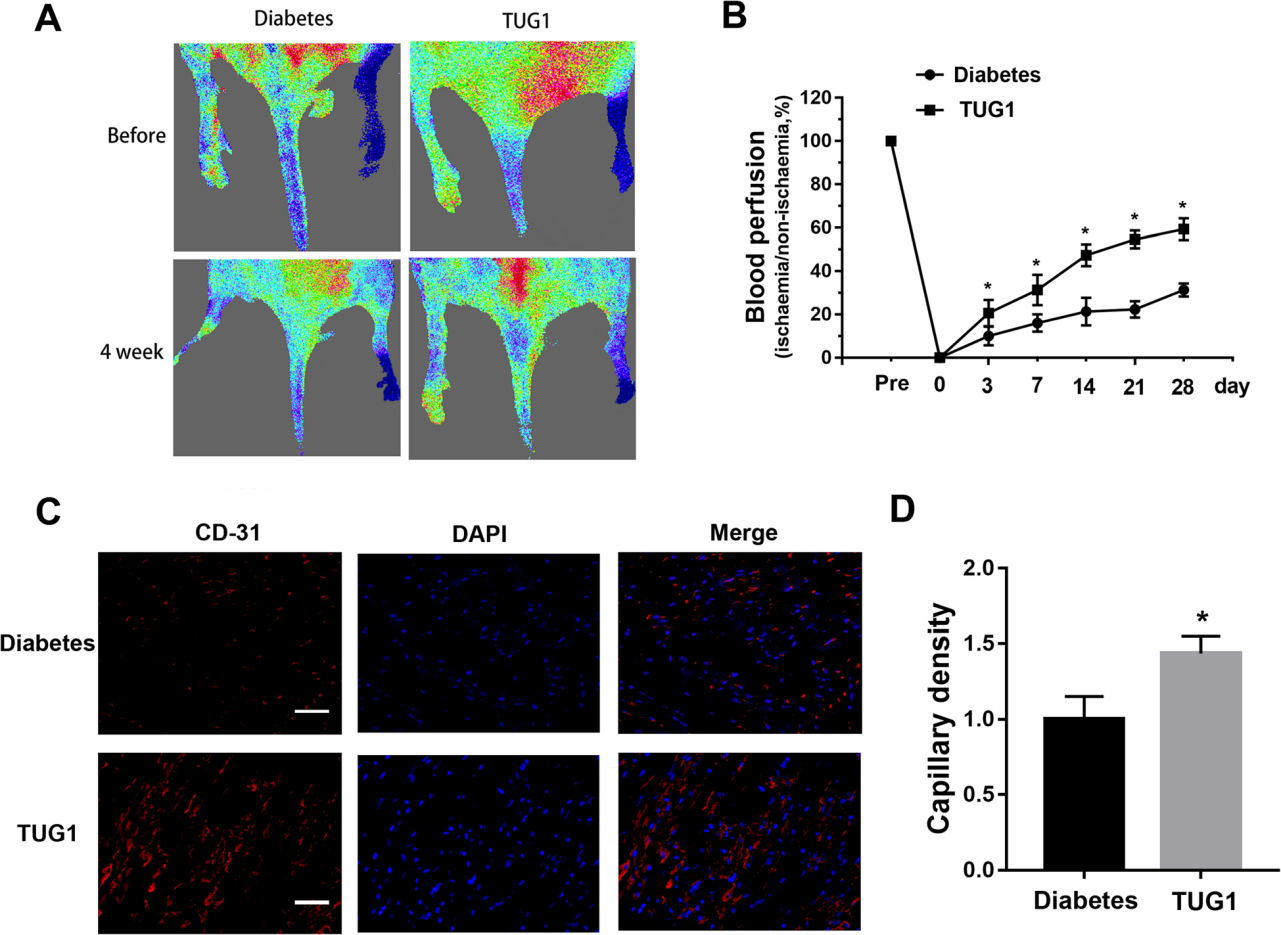
TUG1 overexpression promotes angiogenesis in diabetic mice. **a** Representative evaluation of the ischemic (right) and non-ischemic (left) hind limbs before and 4 weeks after surgery. In color-coded images, red indicates normal perfusion and blue indicates a marked reduction in blood flow in the ischemic hind limb. **b** Blood perfusion was measured using a Laser Doppler perfusion imager system from prior to the operation to 4 weeks after the operation. *p < 0.05 vs diabetes. **c** CD31 immunostaining of gastrocnemius muscle tissue after 4 weeks of hind limb ischemia. Scale bar = 100 μm. **d** Capillary density as expressed by isolectin-positive capillaries per muscle fiber. *p < 0.05 vs diabetes

Finally, in vivo experiments to examine the effects of TUG1 overexpression in an ischemic limb model in diabetic mice were performed (Fig. 8). We ligated the distal portions of the femoral artery and measured hind limb blood perfusion using a Laser Doppler perfusion imager system. We found that perfusion and the expression of capillary-specific markers were significantly higher in limbs that were injected with TUG1 lentiviruses than contralateral untreated limbs (Fig. 8a). Doppler perfusion measurements were obtained two times per week until 28 days and found that perfusion in TUG1-overexpressing animals increased in a time-dependent manner (Fig. 8b). At 4 weeks, the animals were euthanized and we sampled the gastrocnemius muscle tissues of new capillary formation. Evidentially, CD31 immunostaining of gastrocnemius muscle tissue in TUG1-overexpressing animals was significantly greater than that of control animals (Fig. 8c). Finally, capillary density was measured and found significantly higher in TUG1-overexpressing animals (Fig. 8d). Taken together, these findings suggest that TUG1 overexpression improved perfusion in the limbs of diabetic mice.

## Discussion

Type 2 diabetes, if uncontrolled, can lead to many complications, including micro- and macro-angiopathy [30]. Furthermore, as an extension, healing difficulties and neuropathy contribute to the pathology of DFS. Post-natal development of blood vessels post-injury was originally thought to occur through angiogenesis, i.e., migration and differentiation of mature endothelial cells, but recent studies have confirmed the role of EPCs and CPCs in post-natal neovascularization [31, 32]. Evidently, in multiple diabetic models, EPC levels have decreased significantly due to decreased proliferation and increased apoptosis of progenitors in hyperglycemic environments [10–12]. However, the molecular mechanism behind the dysfunction of EPCs is not clear. In this study, we found that TUG1 is highly downregulated in cells grown in high glucose environments. Further, we confirmed that TUG1 stimulates angiogenesis by regulating miR-29c-3p/PDGF-BB/Wnt signaling. To the best of our knowledge, our study is the first to elucidate the mechanism of action of TUG1 in the regulation of angiogenesis by EPCs in the context of the high blood glucose level characteristic of diabetes mellitus.

lncRNAs play a key role in various diseases including diabetes mellitus [33]. In cardiovascular research, lncRNAs have been associated to have a regulatory role in endothelial function. For instance, in vitro studies on umbilical vein endothelial cells have identified non-coding RNA such as MALAT1, LINC00323 [34], and H19 [35] to be regulated under hypoxic environments. Interestingly, they also seem to regulate endothelial cell proliferation, migration, and differentiation. However, there is a need to understand the complex mechanisms interlinking lncRNA to EC dysfunction. TUG1 is frequently referred to as an oncogene [36]. This may be due to the fact that its function in various tissues appears to be the promotion of angiogenesis, an essential process for the growth of solid tumors. In osteosarcoma cells, TUG1 expression is associated with tumor migration and invasion [37]. Interestingly, in the context of the present study, in bladder cancer cells, TUG1 expression was associated with increased proliferation, migration, and invasion [38]. The mechanism appeared to involve binding with miRNA 29c (miR-29c). Similarly, Guo et al. reported that TUG1 promotes BC cell proliferation, migration, and invasion by inhibiting miR-29c [38]. Cai et al. found that TUG1 enhanced tumor-induced angiogenesis and vascular endothelial growth factor (VEGF) expression by inhibiting miR-299 [39]. Lei et al. suggested that TUG1 increases thyroid cancer cell progression, by increasing the tumor cell migration by targeting miR-145 [36].

The endothelial cell dysfunction associated with diabetes mellitus may be thought of as the inverse of the accelerated angiogenesis that characterizes tumor growth and metastasis. Indeed, TUG1 expression was found to be decreased in the context of high glucose levels in a model of diabetic nephropathy [40]. Further, our results are consistent with those of Zang et al. in the sense that the effects of TUG1 expression appeared to be antagonistic to the effects of elevated glucose concentrations with respect to endothelial cell function. Yan et al. reported increased TUG1 expression in human umbilical vein endothelial cells (HUVECs) exposed to high glucose levels [41]. In their study, the investigators found that an inhibitor of the Wnt signaling pathway reversed the proliferative effects of TUG1 on HUVECs, suggesting that the Wnt signaling pathway was involved in TUG1-mediated stimulation of endothelial cell proliferation. A study by Wu et al. on hypoxia-induced myocardial injury concluded that activation of the Wnt/β-catenin signaling pathway mediated the effects of TUG1 [27]. All of these previous studies are consistent with our findings of a regulatory relationship between TUG1 expression and the Wnt signaling pathway.

PDGF-BB is a well-known stimulator of EPC function [42, 43]. We found that PDGF-BB expression levels were lower in the presence of high glucose-containing medium and that overexpression of TUG1 by EPCs reversed this effect. Takahashi et al. found that there was substantial crosstalk between the PDGF-BB and Wnt signaling pathways [44]. Specifically, the authors showed that the effect of PDGF-BB on vascular smooth muscle growth and development was mediated by the beta-catenin component of the wnt signaling pathway. We showed that expression levels of both PDGF-BB (at the mRNA level) and Wnt/β-catenin (at the protein level) were inhibited in the presence of high glucose, supporting the notion that PDGF-BB and the Wnt signaling pathway are involved in mediation of the effects of hyperglycemia on the function of EPCs.

Kamprom et al. studied angiogenic factors secreted by vascular smooth muscle cells and found that PDGF in particular was an important stimulator of angiogenesis in EPCs [45]. Their findings suggest that the effects of hyperglycemia on angiogenesis may be mediated, at least in part, by the regulation of factors that are produced by cells in the mesenchymal lineage, in addition to acting directly on EPCs.

Autophagy, or the lysosome-mediated degradation of cellular contents, is also involved in the regulation of angiogenesis. Several lines of evidence suggest that suppression of autophagy mediates the effects of stimuli that give rise to angiogenesis, including hypoxia, among other exogenous stressors [46]. In the context of our findings, it appears that upregulation of autophagy occurs in the presence of overexpression of TUG1, giving rise to the greater functional activity of EPCs. Recent pieces of evidence indicate that overexpression of miR-29c-3p inhibited autophagy in ovarian cancer [47], and DL-3-nbutylphthalide protected vascular smooth muscle cells from PDGF-BB-stimulated proliferation by inducing autophagy through suppression of the β-catenin signaling pathway [48]. In our study, we speculate that miR-29c-3p/PDGF-BB/Wnt signaling pathway may involve in the autophagy in EPCs under high glucose treatment, but it needs to be further explored in the following research.

We found that the effects of TUG1 appeared to be mediated by the autophagy pathway. Jin et al. found that autophagy was involved in the mechanism of protection of EPCs in diabetic mice [25]. Li et al. studied another lncRNA (WTAPP1) and found that its effects on EPC function were also mediated, at least in part, by the autophagy pathway [49]. Furthermore, Zhou et al. found that the survival of transplanted EPCs in an ischemic limb model was promoted by hypoxia-induced autophagy [50]. Taken together, these data suggest that lncRNA TUG1 enhances the function of high glucose-treated EPCs by regulating miR-29c-3p/PDGF-BB/Wnt signaling, which likely also involves the autophagy pathway.

## Conclusion

Our study found that EPC migration, invasion, and tube formation declined after treatment with high glucose, but improved with TUG1 overexpression. Also, in vivo experiments verified this result in which injection of TUG1 lentivirus in a diabetic mouse ischemic limb model stimulated angiogenesis. Moreover, TUG1 regulates the PDGF-BB/wnt pathway and function of high glucose-treated EPCs via miR-29c-3p. In summary, we report that TUG1 restores high glucose-treated EPC function by regulating miR-29c-3p/PDGF-BB/Wnt signaling.

## Supplementary information

**Additional file 1 Fig. S1.** The expression of TUG1 in EPCs under normal and high glucose conditions. *p <0.05 vs control.

**Additional file 2 Fig. S2.** The expression of NC and TUG1 overexpression transfection in EPCs. *p <0.05 vs NC.

**Additional file 3 Fig. S3.** The expression of NC and miR-29c-3p mimic transfection in EPCs. *p <0.05 vs NC.

## Data Availability

The data that support the findings of this study are available from the corresponding author upon reasonable request.

## Abbreviations

CPCs: Circulating progenitor cells
CQ: Chloroquine
DAPI: 4′,6-Diamidino-2-phenylindole
DFS: Diabetic foot syndrome
Dil-Ac-LDL: Human Dil-Acetylated Low Density Lipoprotein
EPCs: Endothelial progenitor cells
FITC-UEA-1: FITC-labeled *Ulex europaeus* agglutinin 1
HUVECs: Human umbilical vein endothelial cells
TUG1: lncRNA taurine upregulated gene 1
PDGF-BB: Platelet-derived growth factor-BB
PFA: Paraformaldehyde
VEGF: Vascular endothelial growth factor
