# Supplementary figures and images for: TUG1 enhances high glucose-impaired endothelial progenitor cell function via miR-29c-3p/PDGF-BB/Wnt signaling
Source: Stem Cell Res Ther. 2020 Oct 15;11:441. doi: 10.1186/s13287-020-01958-3 (PMC7558752; doi:10.1186/s13287-020-01958-3)

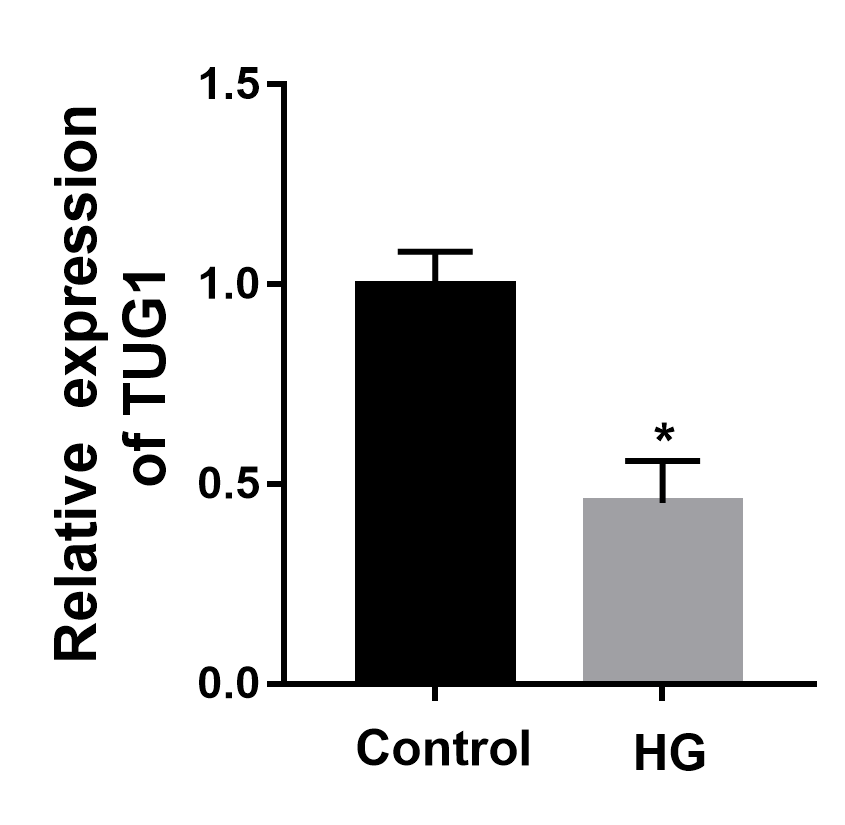

Supplement: Supplementary file 1 — Additional file 1 Fig. S1. The expression of TUG1 in EPCs under normal and high glucose conditions. *p <0.05 vs control. [file 13287_2020_1958_MOESM1_ESM.tif]

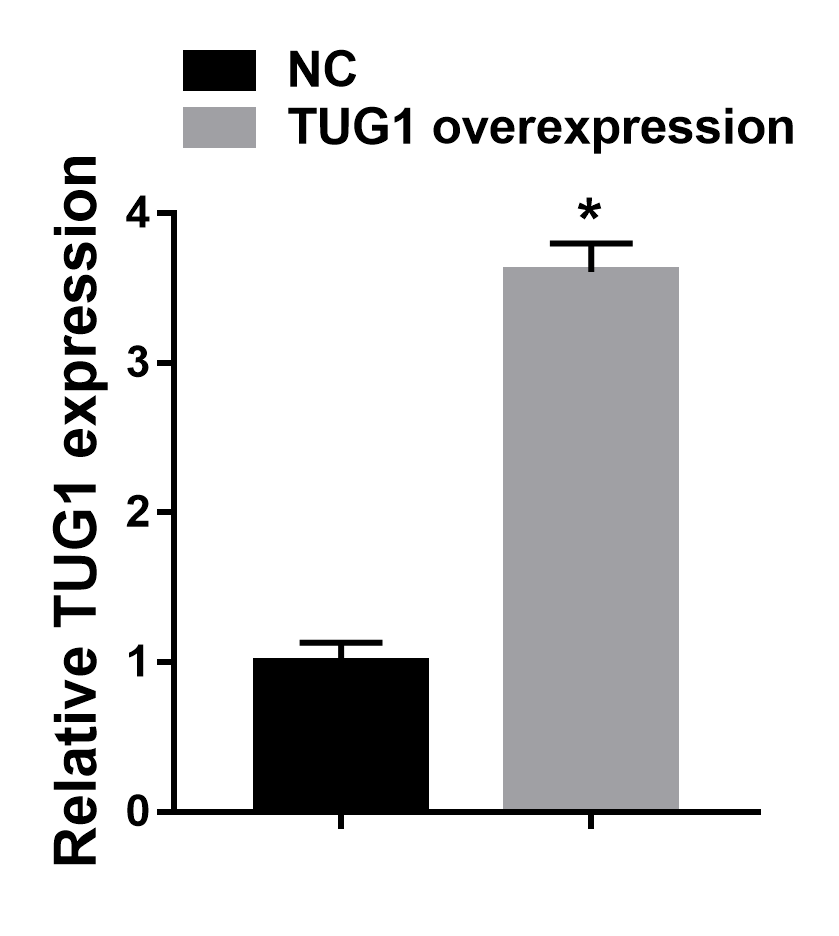

Supplement: Supplementary file 2 — Additional file 2 Fig. S2. The expression of NC and TUG1 overexpression transfection in EPCs. *p <0.05 vs NC. [file 13287_2020_1958_MOESM2_ESM.tif]

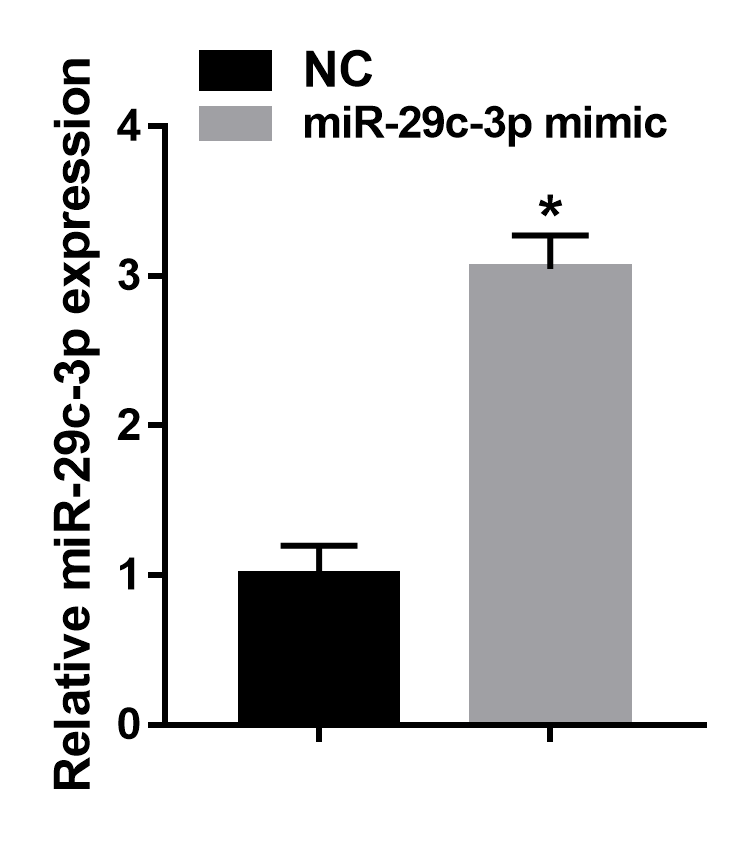

Supplement: Supplementary file 3 — Additional file 3 Fig. S3. The expression of NC and miR-29c-3p mimic transfection in EPCs. *p <0.05 vs NC. [file 13287_2020_1958_MOESM3_ESM.tif]
